# HSV-2 Cellular Programming Enables Productive HIV Infection in Dendritic Cells

**DOI:** 10.3389/fimmu.2019.02889

**Published:** 2019-12-06

**Authors:** Elisa Crisci, Cecilia Svanberg, Rada Ellegård, Mohammad Khalid, Julia Hellblom, Kazuki Okuyama, Pradyot Bhattacharya, Sofia Nyström, Esaki M. Shankar, Kristina Eriksson, Marie Larsson

**Affiliations:** ^1^Division of Molecular Virology, Department of Clinical and Experimental Medicine, Linköping University, Linköping, Sweden; ^2^Department of Pharmaceutics, College of Pharmacy, King Khalid University, Abha, Saudi Arabia; ^3^Division of Experimental Haematology, Department of Clinical and Experimental Medicine, Linköping University, Linköping, Sweden; ^4^Division of Infection Biology and Medical Microbiology, Department of Life Sciences, School of Life Sciences, Central University of Tamil Nadu, Thiruvarur, India; ^5^Department of Rheumatology and Inflammation Research, University of Gothenburg, Gothenburg, Sweden

**Keywords:** HSV-2, HIV-1, HSV-2 and HIV-1 coinfection, dendritic cells, immune responses, DNA sensors

## Abstract

Genital herpes is a common sexually transmitted infection caused by herpes simplex virus type 2 (HSV-2). Genital herpes significantly enhances the acquisition and transmission of HIV-1 by creating a microenvironment that supports HIV infection in the host. Dendritic cells (DCs) represent one of the first innate cell types that encounter HIV-1 and HSV-2 in the genital mucosa. HSV-2 infection has been shown to modulate DCs, rendering them more receptive to HIV infection. Here, we investigated the potential mechanisms underlying HSV-2-mediated augmentation of HIV-1 infection. We demonstrated that the presence of HSV-2 enhanced productive HIV-1 infection of DCs and boosted inflammatory and antiviral responses. The HSV-2 augmented HIV-1 infection required intact HSV-2 DNA, but not active HSV-2 DNA replication. Furthermore, the augmented HIV infection of DCs involved the cGAS-STING pathway. Interestingly, we could not see any involvement of TLR2 or TLR3 nor suppression of infection by IFN-β production. The conditioning by HSV-2 in dual exposed DCs decreased protein expression of IFI16, cGAS, STING, and TBK1, which is associated with signaling through the STING pathway. Dual exposure to HSV-2 and HIV-1 gave decreased levels of several HIV-1 restriction factors, especially SAMHD1, TREX1, and APOBEC3G. Activation of the STING pathway in DCs by exposure to both HSV-2 and HIV-1 most likely led to the proteolytic degradation of the HIV-1 restriction factors SAMHD1, TREX1, and APOBEC3G, which should release their normal restriction of HIV infection in DCs. This released their normal restriction of HIV infection in DCs. We showed that HSV-2 reprogramming of cellular signaling pathways and protein expression levels in the DCs provided a setting where HIV-1 can establish a higher productive infection in the DCs. In conclusion, HSV-2 reprogramming opens up DCs for HIV-1 infection and creates a microenvironment favoring HIV-1 transmission.

## Introduction

Human immunodeficiency virus (HIV) infection is a sexually transmitted disease that reportedly afflicts almost 37 million people globally (1). The risk of contracting HIV-1 infection during a sexual encounter is three-fold higher for individuals infected with genital herpes simplex virus type-2 (HSV-2) (2, 3), and the greatest risk is seen in individuals that have encountered HSV-2 recently (4). Many HSV-2 seropositive individuals are unaware of their infection status, and viral shedding and epithelial micro ulcers are known to occur frequently in asymptomatic individuals. Furthermore, evidences point to that antiviral treatment is unable to suppress HSV-2 infection completely (5–7). The presence of both HIV-1 and HSV-2 creates an environment where immune cells such as dendritic cells (DCs) and Langerhans cells (LCs) can be exposed to and conditioned by either viruses or virus-derived factors. Of interest, the presence of both HSV-2 and HIV-1 appears to enhance HIV DNA levels several fold in DCs (8), LCs (9), and macrophages (10).

The augmentation of HIV infection by the presence of HSV-2 appears to have multiple explanations, including a microenvironment conditioned by the infiltration and persistence of HSV-2-specific CD4+ T cells in the mucosa (11). CD4+ T cells due to their α4β7+ expression are particularly susceptible to HIV infection (12–14). In addition, the increased infiltration of myeloid DCs and plasmacytoid DCs (12) likely contributes to enhancement of the mucosal infection by the recruitment of additional HIV target cells. The reason behind the enhanced infection, besides the higher number of immune cells present, could be due to the activation of DCs located in the mucosa (15). The activated DCs produce high levels of TNF, which conditions the microenvironment as well as adjacent DCs and T cells, rendering them more susceptible to HIV-1 infection by increasing their CCR5 expression (8). In addition, HSV-2 could also precondition DCs and T cells by providing pathogen-associated molecular patterns (PAMPs), resulting in enhanced HIV infection.

The microenvironment at the site of infection is enriched with an array of soluble factors that are likely to influence HIV-1 and HSV-2 infection including components of the complement system (16–18). HIV-1 virions are protected from the normal complement mediated lysis due to the incorporation of complement inhibitory molecules, such as CD55 and CD59 into their lipid membrane (19), which leads to HIV-1 particles opsonized by inactivated complement fragments such as iC3b and C3d (20–22). In addition, the virions transferred during sexual transmission should be complement and/or complement antibody opsonized particles seeing the exposure to seminal fluid and or cervix secretions (19). Both viruses are believed to exploit this arm of the innate immune machinery to enhance the severity of infection and evade complement-mediated lysis of the virions (18, 20, 21, 23).

The activation of innate immunity is an essential component of antiviral defense mechanisms. So far, considerably less is known about the innate cellular programming generated by HSV-2 in DCs that is likely to augment HIV infection. Both HSV-2 and HIV are recognized by an array of pattern recognition receptors (PRRs) in the host. Toll-like receptor (TLR) 2, TLR3, TLR9, and certain intracytoplasmic sensors such as absent in melanoma 2 (AIM2), interferon gamma inducible protein 16 (IFI16), and melanoma differentiation-associated protein 5 (MDA5) have previously been shown to recognize HSV in different mouse and/or human cell types (24, 25). Early HSV-1 recognition in human primary cells, e.g., macrophages, involves two intracellular nucleotide sensing pathways for the induction of type I IFNs and inflammatory factors. With the MDA5/MAVS being paramount for the induction of type I IFNs, whereas the TLR2 pathway appears to play a less important role in early innate responses (24). Recent findings have established the involvement of innate sensors IFI16, and cGMP-AMP synthase (cGAS) in host defense against DNA viruses such as HSV (26). IFI16 sensing of HSV DNA activates the inflammasome machinery and IFN-β responses in some cell types (26). In HSV-1-infected fibroblasts, HSV-1 DNA is sensed by both IFI16 and cGAS (27). IFI16 and cGAS stimulate the STING pathway resulting in activation of TBK1 and IRF3, which leads to IFN-β production (28). HIV-1 is recognized by TLRs, RIG-1-like receptors, NOD2, IFI16, and cGAS (29–31). We have found that signaling in HIV-infected human monocyte derived DCs involves the TLR8 pathway with subsequent activation of the p38 MAPK, ERK, NF-κB, IRF1, and IRF7 signaling cascades, whereas complement-opsonized virions had the ability to suppress these and activate the Lyn, PI3K, IRF3 signaling pathways (20).

In light of the elevated HIV infection and viral shedding in the genital mucosa in HSV-2 seropositive individuals (2, 3, 5–7), and the fact that DCs are targeted by HIV-1 during sexual transmission, we aimed to decipher HSV-2 induced cellular programming underlying the enhanced HIV-1 infection of DCs. Preconditioning of human DCs with HSV-2 prior to HIV-1 exposure elevated the inflammatory and antiviral responses as well as the level of HIV infection in the DC. We found that multiple signaling pathways were activated at the transcriptional level in both free and complement opsonized HSV-2/HIV-1 exposed DCs. The enhancement in HIV-1 infection was dependent on the activation of the STING pathway and required the recognition of structurally intact HSV-2 DNA. The HSV-2 conditioning, by both free and complement opsonized, in the dual exposed DCs decreased the protein levels of IFI16, cGAS, TBK1, and IRF3, all involved in the STING pathway. In addition, the activation of the STING pathway in HSV-2/HIV-1 infected DCs led to decreased levels of several HIV restriction factors, e.g., SAMHD1, TREX1, and APOBEC3G, which diminished their cellular restriction of HIV-1 infection in the DCs. Our work demonstrated that HSV-2 mediated reprogramming enhances HIV's ability to infect the DCs and induces a favorable microenvironment for HIV infection and transmission.

## Materials and Methods

### Reagents

DC culture medium RPMI1640 (GIBCO, Sweden) was supplemented with 2 mM glutamine, 20 μg/mL gentamicin (GIBCO), 10 mM HEPES (GIBCO), and 1% human plasma. Recombinant human GM-CSF (Genzyme) (100 U/mL) and 300 U/mL recombinant human IL-4 (R&D Systems, Minneapolis, MN, USA) were utilized for the *in vitro* propagation of DCs.

### Monocyte-Derived DCs and THP1 Cell Culture

Whole blood from healthy volunteers or buffy coats from the blood bank at Linköping's University Hospital were collected (Ethical Permits M173-07, and M75-08/2008). Peripheral blood mononuclear cells (PBMCs) were separated by density gradient centrifugation using Ficoll-Hypaque (Amersham Pharmacia Biotech, Piscataway, NJ, USA) and incubated on cell culture dishes (BD, Europe) for 1 h at 37°C to allow adherence of DC progenitors and to be able to discard non-adherent cells. Progenitors were differentiated into immature monocyte-derived DCs (henceforth referred to as immature DCs) by adding 100 U/mL GM-CSF and 300 U/mL IL-4 at day 0, 2, and 4 of culture. The DCs were thereafter assessed for expression of CD14 and CD83 markers as a quality control before use in the experiments. In some experiments either wild type THP1 or THP1-Dual™ KO-STING cells (Invivogen, France) were used. The THP1 cells were cultured according to the manufacturer's instructions, activated using phorbol 12-myrisate 13-acetate (PMA, 10 μg/mL) and incubated 2 days before the cells were infected and treated in the same manner as described below for DCs.

### Virus Propagation and Titration

HSV-2, virus stock was prepared in African green monkey kidney (GMK) cells cultured in DMEM supplemented with 10% heat inactivated (HI) FCS as described previously (32). The HSV-2 strain 333 was used either as infectious, or as γ-irradiated (30 min) inactivated virus. HIV-1BaL/SUPT1-CCR5 CL.30 (lot 4235, 4238, 4313, and 4366) was produced using chronically-infected cultures of the ACVP/BCP cell line (No. 204), originally derived by infecting SUPT1-CCR5 CL.30 cells (generously gifted by Dr. J. Hoxie, University of Pennsylvania) with an infectious stock of HIV-1BaL (NIH AIDS Research and Reference Reagent Program, Catalog No. 416, Lot No. 59155). Virus was purified and concentrated as previously described (33) and aliquots were frozen down. All virus preparations were assayed for infectivity.

### Generation of GFP Reporter CCR5-Tropic Virus

NLENG1-IRES proviral construct was used to generate NLENG1-IRES-70 by replacing ENV with YU-2 ENV as described elsewhere (34, 35). The proviral construct was generously donated by Dr. David N Levy (New York University, New York, NY, United States). HEK-293T cells were cultured in DMEM containing 10% HI FCS, and at ~70% confluency, the cells were transfected with NLENG1-IRES-70 proviral construct using the CaPO_4_ method. After 8 h of transfection, the media was replaced with DMEM supplemented with 1% HI FCS. The GFP-HIV was harvested the next day by collecting supernatant, and cell debris were removed by pelleting at 2,500 rpm for 5 min. Virus stocks were aliquoted and frozen at −80°C.

### Opsonization of HSV-2 and HIV

Complement opsonization of HSV-2, HIV, and GFP-HIV was done by incubation of the virions with an equal volume of human serum (HS) (Ethical Permits M173-07, and M75-08/2008). Different HS were used for opsonization; HSV-1 and HSV-2 seronegative HS was used to opsonize HSV-2 virus (referred as CHSV), and HIV and HSV-2 seronegative HS was used to opsonize HIV-1 or GFP-HIV-1 viruses (referred to as CHIV and CHIV-GFP). The HS was tested for HSV antibodies using HerpeSelect® 1 ELISA IgG and HerpeSelect® 2 ELISA IgG kits (Focus Diagnostics, Cypress, CA, USA). Free viruses HSV-2, HIV-1, and GFP-HIV-1 (referred to as HSV, HIV, and GFP-HIV) were diluted to the same concentration opsonized virus. The negative control, i.e., mock, was treated with culture medium. All virus groups were incubated for 1 h at 37°C and used in the co-infection experiments.

### HSV-2 and HIV Infection of DCs

Immature DCs (10^6^ cells/mL) were infected with mock, (30 ng/μl) HIV, or CHIV as single infection. HIV/HSV or CHIV/CHSV were used in two setups: direct and delayed co-infection. In the delayed group, 1 MOI HSV-2 and CHSV were added to DCs for 2 h and the cells were thereafter washed before the addition of HIV and CHIV for 4, 6, or 22 h. In the direct co-infection setup, both virus types were added to the DCs simultaneously for 3 h, washed and then incubated for additional 21 h in all the set ups DCs were cultured in 1% human plasma medium. The DCs were harvested, washed, and lysed with Bioline RLY lysis buffer (Bioline, UK) for RNA extraction, or fixed with 4% paraformaldehyde (PFA) 10 min at 4°C for immune phenotyping by flow cytometry.

In order to examine the level of productive HIV infection, the two setups of co-infection were performed, and supernatants were collected on day 1, 4, 7, and 10. After collection of the supernatant, the cells were re-suspended in 10% FBS before addition of IL-4 and GM-CSF. HIV p24 level was determined by an in house p24 ELISA assay (36). Additionally, HIV productive infection was assessed using a GFP reporter CCR5-tropic virus using the same experimental set up as described earlier for the other viruses. DCs were infected with GFP-HIV for 3 and 5 days before the percentage of positive GFP cells were analyzed by flow cytometry.

### Total RNA Extraction, Reverse Transcription, and qPCR

Total RNA from DCs exposed to the different conditions was extracted using a commercial Isolate II RNA Mini or Micro Kit (Bioline, UK), and total cDNA was produced by SuperScript III Reverse Transcriptase First Strand cDNA Synthesis kit (Invitrogen, Carlsbad, CA, USA). Quantification of gene transcripts was performed using the SensiFAST SYBR® Hi-ROX Kit (Bioline, UK) and CFX96 Touch Real-Time system (BIO-RAD Inc.). Primers targeting β-actin and GAPDH were used as housekeeping genes for reference as described by others (37). Primers were procured from CyberGene AB (Stockholm, Sweden). To compensate for variation between plates, values were normalized as previously described (38).

### ELISA and Cytometric Bead Array

The levels of TNF (Mabtech, Sweden) and IFN-β (VeriKine kit; PBL Assay Science, USA) proteins were assessed in DC culture supernatants after ca 20 h of infection by ELISA according to the manufacturers' protocols. Cytometric bead arrays (BD Biosciences, Stockholm, Sweden) were used to measure the levels of cytokines and chemokines in cell supernatants and for measuring the levels of phosphorylated protein in DCs lysates (BD Biosciences, Stockholm, Sweden) according to the manufacturer's protocols.

### Flow Cytometry

The quality of immature DCs was assessed by staining with anti-human CD83 and CD14 PE-conjugated antibodies (BD, Europe). DCs were used if the purity was >95% and their expression of CD14 and CD83 were <10%. The expression of HSV-2 antigens in HSV-2 exposed DCs was done by a polyclonal anti-HSV-2 antibody (B0116, Dako, Denmark). The effects infection had on various proteins were evaluated by staining with PE-Cy7-conjugated anti-human APOBEC3G (Abcore, USA), Alexa Fluor 647-conjugated anti-human SAMHD1 (Bioss Inc. USA), Alexa Fluor 647-conjugated anti-human TREX1, anti-human IRF3, PE-conjugated anti-human NAK/TBK1, anti-human IFI16 (Abcam, UK), anti-human CF150 (cGAS) (Thermofisher Scientific), and PE-conjugated anti-human STING antibodies (BD Europe). The unconjugated antibodies were stained with conjugated secondary anti-rabbit antibodies (DAKO). The stained DC samples were assessed by flow cytometry (FACS Canto II, BD) and analyzed by FlowJo (Treestar, Ashland, OR, USA).

### Ligands and Inhibitors

The following TLR ligands and inhibitors were used in the experiments: TLR2 ligand PAM (5 μg/mL), TLR3 ligand Poly I:C (40 μg/mL), 2′3′-cGAMP, (10 μg/mL), Poly dA:dT (0.5 μg/mL), HSV-60 Naked DNA (10 μg/mL), oligonucleotide A151 ODN TTAGGG (1–3 μM) (Invivogen, France), human pp65 (UL83) recombinant protein (10–15 μg/mL) (Miltenyi Biotec, Sweden), recombinant human papillomavirus type 18 protein E7 (10–20 μg/mL) (SMS-gruppen, Denmark), and acyclovir (20–50 μM) (SIGMA). Acyclovir was added 1 h before addition of virus, incubated with the virus for 2 h, washed away, replenished and thereafter kept in the culture throughout the incubation. Intracellular delivery of cGAMP, Poly dA:dT, HSV-60 Naked DNA, A151 oligonucleotide, UL83 protein, and E7 protein was achieved using a commercial DOTAP system (SIGMA). Briefly, the different ligands and inhibitors were mixed with HEPES buffered saline and DOTAP for 30 min at room temperature, incubated for 2 h at 37°C with DCs, and washed prior to infection. DOTAP alone served as a control for the effect of the delivery system.

### RNAseq

RNA (5 ng) was subjected to whole transcriptome amplification using NuGEN's Ovation RNA-Seq V2 kit (San Carlos, CA, USA) according to manufacturer's instructions. Briefly, cDNA was amplified from total RNA using a single primer isothermal amplification (SPIA). The amplified cDNA samples were subsequently purified using a MinElute Reaction Cleanup Kit (Qiagen; Valencia, CA, USA). The cDNA samples were fragmented into smaller pieces, blunt-ended, and ligated to indexed (barcoded) adaptors and amplified using an Ultralow System V2 kit according to the manufacturer's protocol. Final library size distribution was determined using an Agilent Bioanalyzer 2100. Five libraries from five different donors were sequenced on the Illumina NextSeq500 platform (San Diego, CA, USA). The FASTQ files were uploaded to UPPMAX and quality checked using fastQC (39). Trimmomatic (40) was used to remove adaptors and low-quality bases and the reads were then mapped to human reference genome hg19 using STAR (41). Counts for each gene were calculated using featureCounts (42). The data was normalized and differentially expressed genes determined using R/DeSeq2 (43). Analysis of pathways was done by Ingenuity Pathway Analysis (Qiagen), R analysis, Gene Ontology (GO) Enrichment Analysis (Geneontology.org), and custom gene lists.

### Statistical Analysis

GraphPad Prism 5 (GraphPad Software, La Jolla, CA, USA) was used for the analysis of all data except RNAseq. Repeated measures ANOVA followed by a Bonferroni post-test or a two-tailed paired *t*-test were used to test for statistical significance. Results were considered statistically significant if *p* < 0.05. All experiments were performed a minimum of four times using cells derived from different blood donors. When experimental values were normalized, the mean of free virus or mock were set to 1. qPCR results were normalized for variation between plates as previously described (38). In brief, each value was subtracted by the average of all values and then the obtained values were divided by the average of mock or free virus depending on the different experiments.

## Results

### HSV-2 Conditioning of Human DCs Rendered Them More Susceptible to HIV Infection

**Figure 1:**
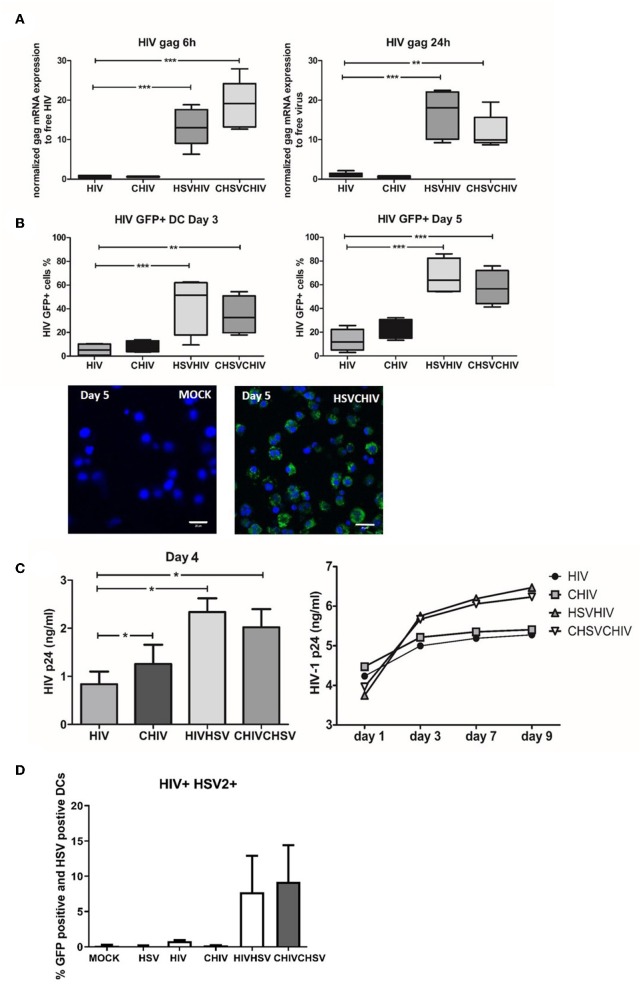
HSV-2 conditioning of human dendritic cells rendered them more susceptible to HIV productive HIV infection. Dendritic cells were exposed to free HSV-2 (HSV) or complement opsonized virus (CHSV) for 2 h then washed and infected with free HIV, either HIV-1 BaL or GFP tagged CCR5 tropic HIV-1(GFP-HIV) or complement opsonized HIV (CHIV) for 6 or 22 h. HSV-2 positive DC was measured using a polyclonal anti-HSV-2 Ab followed by fluorochrome conjugated secondary Ab **(A)** mRNA expression levels of HIV gag were determined by qPCR at 6 and 24 h. Data are normalized to free virus, which is set as one. **(B)** GFP-HIV positive cells were evaluated by flow cytometry after 3 and 5 days post-HIV infection or by confocal microscopy (40×) 5 days post-infection. GFP-HIV productively infected cells (green) stained with a nuclear staining with DAPI (Blue). Size bar = 20 μm. **(C)** HIV-1 p24 release by productively infected DCs were determined by ELISA from day 1–9 post-HIV infection. **(D)** Percentage of HIV GFP positive and HSV-2 antigen positive DCs measured by flow cytometry 24 h post-infection. *p < 0.05; **p < 0.005; ***p < 0.0005. *N* = 5–8.

It is now evident that HSV-2 infection of the genital tract predisposes for HIV infection and increased viral shedding at the mucosal site (2, 3, 5–7). Nevertheless, the mechanisms underlying the elevated infection remain ambiguous. Here we evaluated the role of DCs in the enhanced infection by assessing the effects pre-exposure to HSV-2 exerted on HIV infection of human monocyte-derived DCs, and the role of opsonization of the viruses with complement. The levels of HIV gag mRNA transcripts were significantly enhanced in HSV/HIV dual exposed DCs compared to the levels induced by HIV alone at both 6 and 24 h (Figure 1A). The HSV-2 associated enhancement of HIV infection is in accordance with the findings by Marsden et al. (8). Moreover, the DCs exposed to complement opsonized HSV-2 gave a higher tyrosine kinese (TK) in both dual and single exposed DCs compared to free virus as shown previously, but there was no alteration in the HSV-2 infection when measured by TK expression in the HIV/HSV dual exposed DCs compared to HSV-2 single exposed DCs (Supplementary Figure 1). Productive HIV infection was also significantly elevated in HSV/HIV dual exposed cells as measured by the percentage of GFP-HIV positive DCs by flow cytometry and confocal microscopy day 3 and 5 post-exposure (Figure 1B), and the levels of HIV p24 in the culture supernatants day 4 and day 1–9 (Figure 1C). The opsonization of the virions did not significantly change the level of HIV infection in the HSV/HIV infected cells (Figures 1A–C). Of note, the presence of HSV-2 enhanced HIV infection, both when the DCs where preconditioned with HSV-2 before HIV infection and when HSV-2 and HIV were introduced to the DCs simultaneously. Under normal circumstances, only a small fraction of the DCs will be productively infected by HIV (Figure 1B), and the same also applies for HSV-2 (18). Still, all DCs in the cultures are exposed to the viruses and influenced by them either indirectly or directly also in the absence of productive infection (18, 36). The amount of DCs that support the productive infection of both HIV and HSV-2 is low and has been shown by Marsden et al. to represent only 1% of the cells. In our system we determined that the level of DC with productive HIV infection and positive for HSV-2 antigen to be 5% for DC exposed free HIV/HSV and ~12% for DC exposed to complement opsonized CHIV/CHSV (Figure 1D), which could reflect that not all HSV-2 antigen positive cells are productively infected, rather that some cells have an abortive HSV infection (44–46). The data for direct and delayed co-infection gave very similar data so the experiments presented and carried out onwards are from delayed coinfection setup. Taken together, our data clearly demonstrates that exposure to HSV-2 enhances the ability of HIV to productively infect DCs, which directed us to elucidate the underlying mechanism responsible for enhanced HIV infection.

### Distinct Cellular Programming Was Induced in DCs by the Different HIV and HSV-2 Infection Conditions

**Figure 2:**
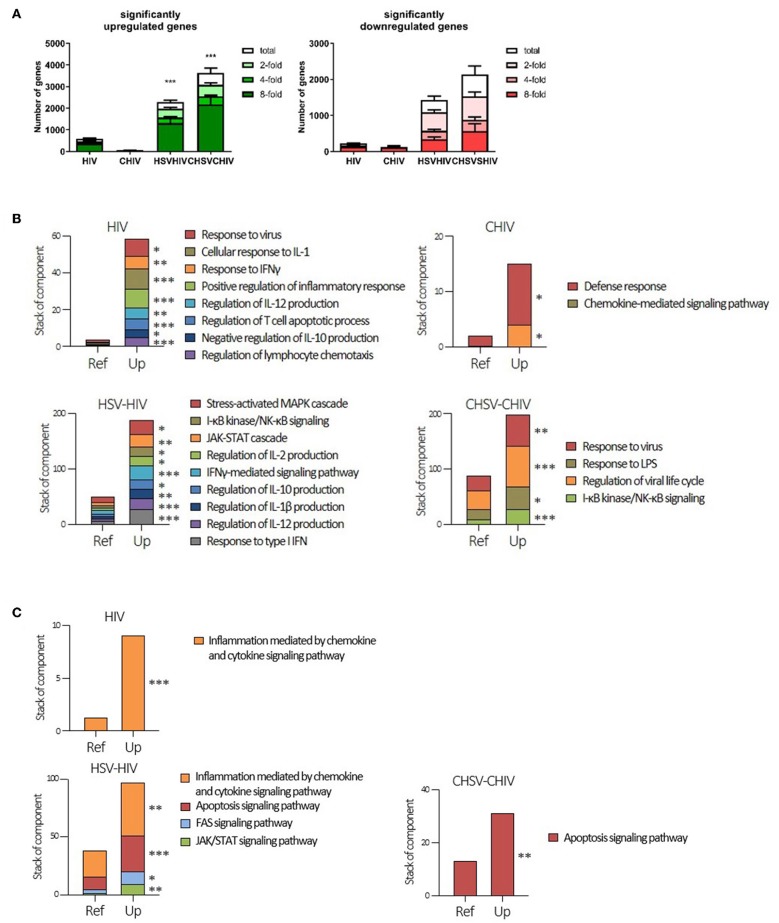
Cellular programing of dendritic cells by HIV single and HIV/HSV dual infection. Dendritic cells were exposed to HSV-2 (HSV) or complement opsonized virus (CHSV) for 2 h then infected with HIV or complement opsonized HIV (CHIV) for 24 h. Whole transcriptome sequencing was performed. **(A)** Analyzes of amount of significantly upregulated or down regulated genes assessing total, two-, four-, and eight-fold changes compared to mock **(B)** Gene enrichment analysis of genes significantly upregulated three-fold or higher with *p* ≥ 0.05 with focus on inflammatory and antiviral pathways/factors. **(C)** Gene enrichment analysis of genes significantly upregulated three-fold or higher with *p* ≥ 0.05. GO enrichment analysis was done with PANTHER pathways data set. Terms with statistical significance in any of gene list are shown as stacked bar graph. *p < 0.05, **p < 0.005, ***p < 0.0005. Y-axis = number of listed genes involving in indicated PANTHER pathway. Ref = expected gene number from reference (whole human genes in database). *N* = 5.

To further understand the cellular programming during HSV/HIV infection, we performed RNA seq, which provided the whole transcriptome profile in the DCs induced by single exposure to HIV vs. dual exposure to HSV-2 and HIV. The transcriptome analysis demonstrated activation and involvement of distinct sets of pathways in the single and dual infected DCs respectively. The dual virus exposed DCs upregulated or downregulated significantly more genes than DCs exposed to HIV and CHIV alone (Figure 2A). This demonstrated that exposure to both HSV and HIV was a more potent activator of DCs than HIV alone, and that complement opsonization of the virions suppressed DC activation. Next, our gene enrichment analysis clearly established that each of the different viral conditions gave rise to unique gene expression patterns within the different signaling pathways (Figures 2B,C; Supplementary Figure 2). The CHIV and the CHIV/CHSV conditions had less enriched pathways compared to the free virus groups (Figures 2B,C). Genes in the interleukin and the JAK/STAT signaling pathways were enriched in both HIV, CHIV, and HIV/HSV dual exposed DCs, but only HIV/HSV had significant enrichment (Supplementary Figure 2). The ubiquitin-proteasome pathway has recently been shown to be important in viral pathogenesis (47). Genes involved in this pathway were significantly enriched in the free HIV and HIV/HSV exposed DCs but only a restricted number of genes were enriched in the complement exposed groups (Supplementary Figure 2). The enrichment analysis of genes with decreased expression levels after the HIV infection revealed that genes in the cholesterol biosynthesis pathway were significantly enriched in CHIV exposed DCs and genes involved in the pyrimidine metabolism were enriched in both HIV/HSV and CHIV/CHSV exposed DCs (Supplementary Figure 2). A more specific, in-depth, analysis of gene enrichment focusing on inflammation, antiviral and immune responses uncovered a pattern of significantly enriched pathways distinctly different for each type of infection condition, although there was some overlap across the groups (Figures 2B,C; Supplementary Figure 2). For instance, upregulated genes in JAK-STAT cascade was significantly enriched for all groups besides CHIV (Supplementary Figure 2).

### Inflammatory Factors Were Highly Elevated in Dual HSV-2 and HIV Exposed DCs

**Figure 3:**
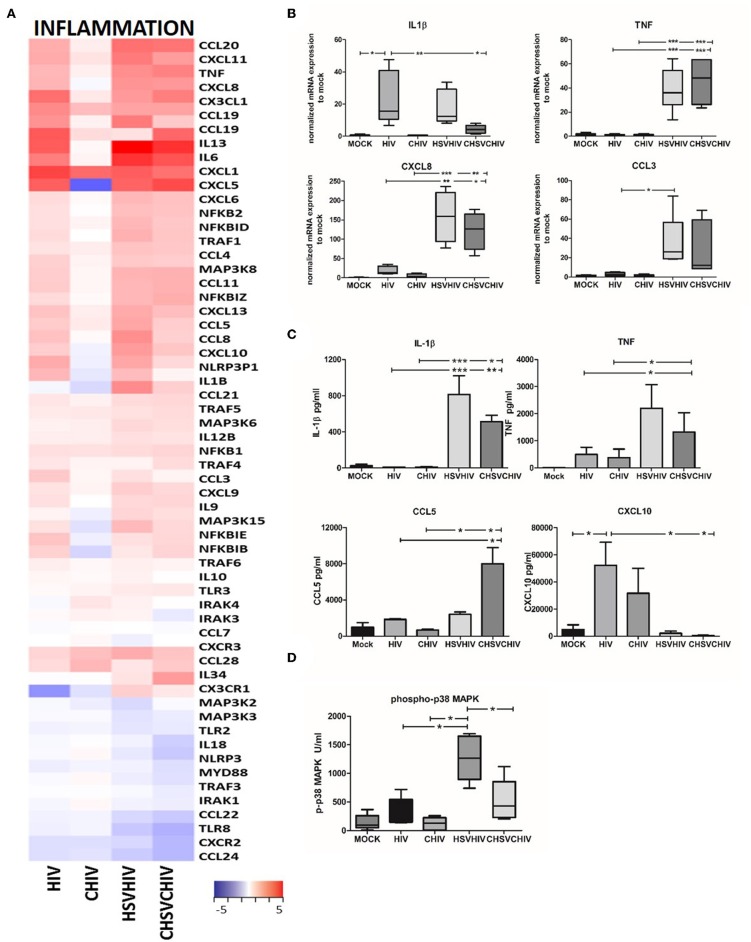
Inflammatory pathways were highly activated in HSV/HIV exposed DCs on the mRNA transcription. **(A)** Dendritic cells were exposed to free HSV-2 or complement opsonized virus (CHSV-2) for 2 h then infected with HIV or complement opsonized HIV (CHIV) for 22 h. Heat map over inflammatory factors significantly up or down regulated in one or several of the HIV, CHIV, HSV-2/HIV, or CHSV-2/CHIV DC groups compare to mock treated DCs from the RNA seq **(B)** mRNA expression levels of IL-1β, TNF, CCL3, CXCL8 were determined by PCR. Data were normalized to mock set as 1. **(C)** Protein levels of secreted cytokines were assessed with ELISA for TNF and cytokine bead array for IL-1β, CCL5, and CXCL10. **(D)** Level of MAPK p38 phosphorylation was assessed in lysates from DCs exposed to HSV-2 or CHSV-2 for 2 h followed by HIV or CHIV exposure for 4 h by phosphoprotein bead array. *p < 0.05; **p < 0.005; ***p < 0.0005. *N* =5–8.

HIV and HSV-2 are both known to induce inflammatory pathways in immune cells with HSV-2 being the more potent activator (18, 20) and our transcriptome analysis clearly demonstrated the activation and involvement of several inflammatory pathways. HSV/HIV or CHSV/CHIV induced distinct gene expression patterns in DCs for inflammatory genes compared to single HIV or CHIV infection (Figure 3A). Moreover, the complement opsonized groups had lower transcription levels of many of inflammatory factors (Figure 3A). HIV alone, HSV/HIV and CHSV/CHIV activated multiple inflammatory factors such as IL-6, TNF, CXCL1, and CCL20, with the highest expression activated in co-infected groups. Complement opsonization lowered the transcription of several factors such as CCL1, CCL5, CCL8, and IL-1β seeing that they were lower in the opsonized groups compared to free virus conditions. CCL21, CCL28, and IL34 were only upregulated in the dual exposed groups. Few genes had higher gene expression levels in the CHIV group and included CXCR3 and IL-10. Next, we verified the expression pattern of some of the inflammatory factors by qPCR and the translation by ELISA or flow-based cytokine bead array. HSV alone was the leading inducer for TNF and CCL3, whereas HIV-1 was more potent for the induction of CXCL8 and IL-1β (Supplementary Figure 3). The HSV/HIV and CHSV/CHIV gave rise to higher mRNA expression levels of TNF, CXCL8, and CCL3 compared to the free and complement-opsonized HIV (Figure 3B). The levels of IL-1β mRNA were similar between in HIV and HIV/HSV exposed DCs (Figure 3B), whereas the protein levels were higher in the dual exposed DCs (Figure 3C). The secretion of TNF, and chemokines CCL3 and CCL5 followed the pattern of mRNA expression and/or transcriptome data (Figures 3A–C; data not shown). The protein levels of other inflammatory factors such as CXCL10 were lower in the DCs exposed to both viruses compared to HIV alone (Figure 3C). These discrepancies between protein levels and the transcriptome data and can be explained by post-transcriptional regulation and/or proteolytic degradation (48–50). The transcription of inflammatory factors is activated by several signaling pathways including the MAPK p38 pathway, and dual exposure significantly enhanced the level of phosphorylated p38 with a trend that opsonization of the virions decreased phosphorylation of p38 (Figure 3D). JNK phosphorylation displayed a similar pattern of activation as MAPK p38 but there were no significant differences (data not shown), which could be due to the time point selected for the measurement.

### HIV/HSV Exposure Induced Higher Expression of Several Antiviral Factors Including IFN-β in the DCs Compared to Exposure With HIV Alone

**Figure 4:**
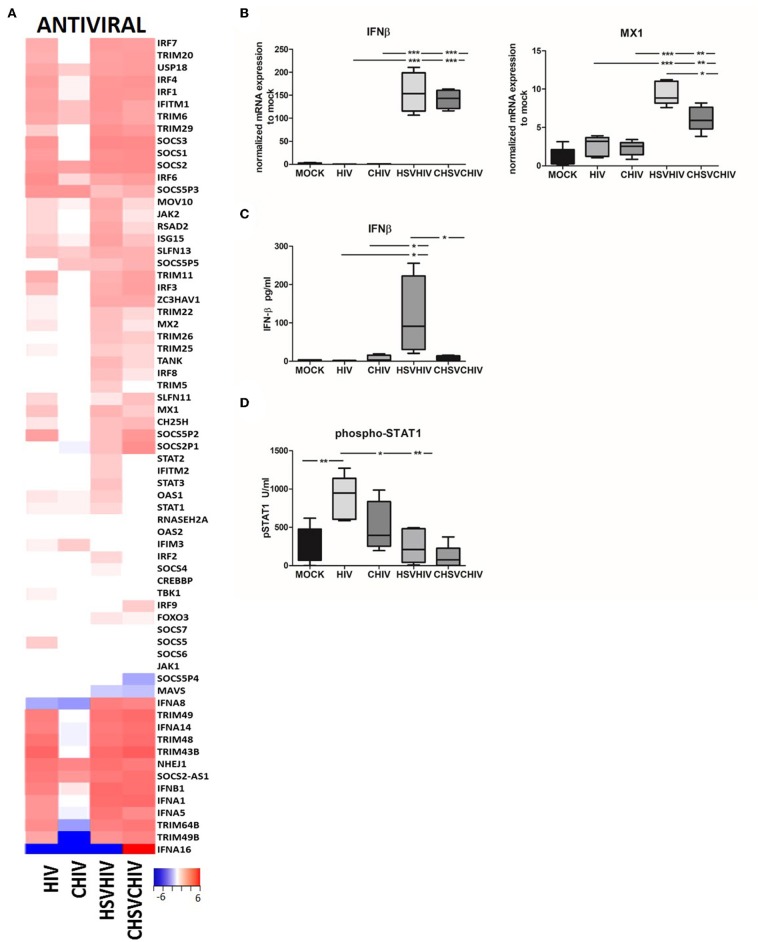
Antiviral factors and pathways were highly activated in HSV/HIV exposed dendritic cells. Dendritic cells were exposed to HSV-2 or complement opsonized virus (CHSV-2) for 2 h followed by HIV or complement opsonized HIV (CHIV) infection for 22 h. **(A)** Heat map from RNA seq data of antiviral factors significantly up or down regulated in one or several of the HIV, CHIV, HSV-2/HIV, or CHSV-2/CHIV infection conditions compared to mock treated DCs **(B)** mRNA expression levels of IFN-β, and MXA were accessed by PCR. Data were normalized to mock set as 1. **(C)** Levels of secreted IFN-β were evaluated by ELISA. **(D)** Level of STAT1 phosphorylation was assessed in lysates from DCs exposed to HSV-2 or CHSV-2 for 2 h followed by HIV or CHIV exposure for 4 h by phosphoprotein bead array. *p < 0.05; **p < 0.005; ***p < 0.0005. *N* = 5–8.

The transcriptome analysis (Figure 2) clearly demonstrated the activation and involvement of an array of antiviral pathways in the DCs exposed to both HIV only as well as both HSV and HIV. Here we assessed factors involved in the activation and regulation of the cellular antiviral defense (Figure 4A). The pattern for many antiviral factors induced in the DCs was similar between HIV, and the dual virus DCs conditions and in general lower level of antiviral factors activated in the complement groups. Several TRIM genes, type I IFN genes, SOCS genes, and IRF genes were upregulated in free HIV and the dual exposed DCs, with the highest transcription levels mostly found in the dual exposed DCs. Many antiviral genes were upregulated in HIV/HSV and CHIV/CHSV exposed DCs, whereas few antiviral genes were only found to be upregulated in HIV exposed DCs and include IFITM3, SOCS5, and TBK1 for free HIV and IFITM3 for CHIV (Figure 4A). Next, we confirmed some of these antiviral factors at the mRNA and protein levels. The single HSV and dual HSV/HIV exposure enhanced the mRNA expression levels of IFN-β and MX1 compared to single exposure to HIV (Figures 4A,B; Supplementary Figure 4), clearly showing that the HSV is the component responsible for the strong antiviral responses. Furthermore, IFN-β secretion was higher in the dual exposed samples, with the highest levels induced by HSV/HIV (Figure 4C). Interestingly, the levels of IFN-β did not negatively correlate to the HIV infection levels suggesting that IFN-β had a restricted impact on the infection even at the high levels of IFN-β seen in dual exposed DCs. The IFN-β feedback loop induces the activation of STAT1 and transcription of IFN-regulated genes (ISG). Our data clearly indicated that the exposure of DCs to HIV induced a significantly higher STAT1 phosphorylation at 6 h compared to CHIV, HSV/HIV, and CHSV/CHIV (Figure 4D). In addition, HIV and HSV/HIV also significantly upregulated several ISGs, including IFITM1, RSAD2, and ISG15 (Figure 4A).

### HSV-2 Augmentation of HIV Infection in DCs Required Intact HSV-2 DNA Structure

**Figure 5:**
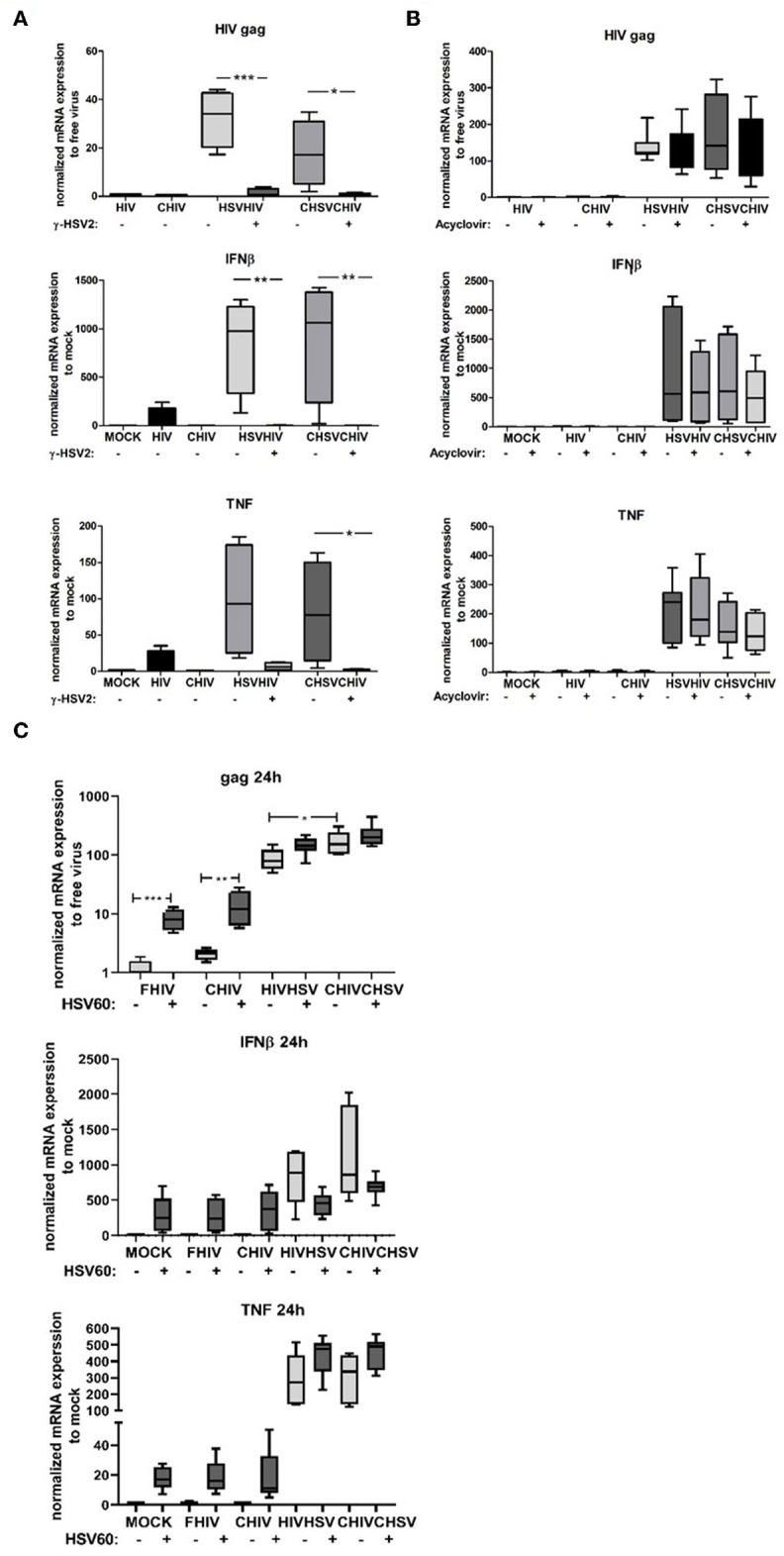
Intact HSV dsDNA is required for the enhanced HIV infection in DCs. **(A)** Dendritic cells were exposed to infectious or gamma irradiated HSV-2 (γ-HSV-2) or complement opsonized virus (CHSV) for 2 h then washed and infected with HIV or complement opsonized HIV (CHIV) for 22 h. mRNA expression levels of HIV-1 gag transcript, TNF, and IFN-β were determined by PCR. **(B)** DCs were pretreated with acyclovir (20–50 μM) for 1 h and then exposed to HSV, or CHSV for 2 h then washed and infected with HIV or CHIV for 22 h with acyclovir replenished to the culture. Gene expression levels of HIV-1 gag transcript, TNF, and IFN-β were determined by PCR. **(C)** DCs were treated with or without HSV60 delivered by DOTAP and thereafter exposed to HSV, or CHSV for 2 h then washed and infected with HIV or CHIV for 22 h with acyclovir replenished to the culture. Gene expression levels of HIV-1 gag transcript, TNF, and IFN-β were determined by PCR. Data were normalized to HIV set as 1 for HIV-1 gag transcript and mock set as 1 for TNF, and IFN-β. *p < 0.05; **p < 0.005; ***p < 0.0005. *N* = 5–8.

To assess if the enhanced HIV infection in the DCs was a consequence of productive HSV-2 infection with full length DNA replication or if it was sufficient with the delivery of intact accessible dsDNA by the virions we used acyclovir and γ-irradiated inactivated HSV-2 (51). In the absence of replication of HSV-2 DNA, the γ-irradiated inactivated HSV-2 failed to elevate the level of HIV infection and the expression levels of TNF and IFN-β (Figure 5A), clearly indicating that the increased HIV infection of DCs is dependent on the presence of accessible and/or intact HSV-2 DNA. The inactivation of HSV-2 by γ-irradiation induces DNA damage that could render the viral DNA less reactive with the intracellular PRRs, affect the transport of the DNA to the nucleus, and/or affect the release of the DNA from the viral capsid. We used the HSV-2 replication inhibitor acyclovir to examine if the presence of intact HSV-2 DNA in the DCs was sufficient for enhanced HIV infection or if active HSV-2 DNA replication was required. The inhibition of active HSV-2 DNA replication in DCs had little effect on the HIV infection and the IFN-β, but decreased the IFN-β response, these results were however not statistically significant (Figure 5B). To further establish the role of dsDNA, we delivered HSV60, a 60 bp dsDNA sequence from HSV-1 using DOTAP into DCs. The intracellular dsDNA significantly enhanced the FHIV and CHIV infection compared to HIV alone (Figure 5C). Taken together, the enhanced HIV infection and increased production of IFN-β in dual exposed DCs required the presence of HSV-2 dsDNA in its natural configuration, which suggests that the activation of intracellular PRRs such as DNA sensors may underlie the HSV-2 enhancement of HIV infection of DCs.

### Intracellular HSV-Derived PAMPs Are the Major Factors Involved in the Enhanced HIV Infection of DCs

**Figure 6:**
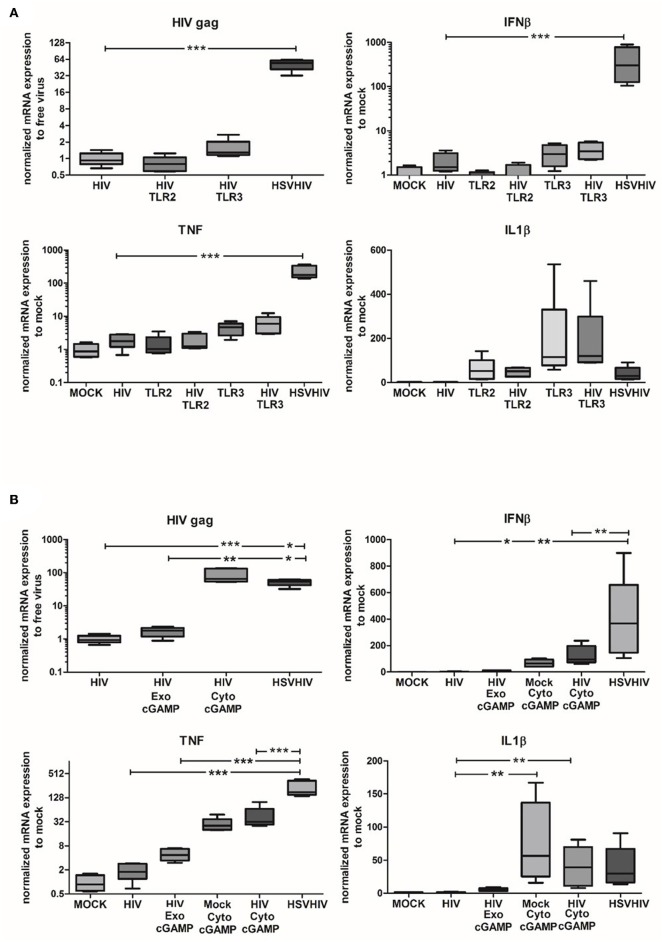
Intracellular HSV-2 derived nucleotides are major factors involved in the enhanced HIV infection in DCs. **(A)** Immature DCs were exposed to TLR2, or TLR3 agonists alone or in combination with HIV for 16 h or exposed to HSV-2 (HSV) for 2 h followed by HIV for 16 h. HIV gag transcript, IL1-β, TNF, and IFN-β expression were evaluated by qPCR. **(B)** cGAMP was given directly or delivered via DOTAP to the DCs alone or together with HIV for 16 h or the DCs were exposed to HSV for 2 h followed by exposure to HIV for 16 h. HIV gag transcript, IL1-β, TNF, and IFN-β expression were evaluated by PCR. HIV gag transcript data were normalized to HIV set as 1 and TNF, IL1-β, IFN-β data were normalized to mock set as 1. *p < 0.05; **p < 0.005; ***p < 0.0005. *N* = 5–8.

To assess the role of different PAMPs in the HSV-2-induced enhancement of HIV infection in DCs, we investigated if TLR2, TLR3, or cGAS/STING activation could mimic the effects seen after HSV-2 infection. Both TLR2 and TLR3 are known to be involved in HSV-2 infection (52, 53). TLR2 stimulation simultaneously with HIV infection did not affect HIV gag expression compared to HIV alone (Figure 6A). Targeting TLR3 gave only a slightly increased HIV gag expression compared to the dramatically elevated HIV gag expression in DCs exposed to both HSV-2 and HIV (Figure 6A). While inflammatory responses to TLR3 agonist alone and TLR3 agonist in combination with HIV were higher than inflammatory responses to HIV alone, neither TNF nor IL1-β approached the levels seen in dual exposed with HSV-2 (Figure 6A; data not shown). Seeing that TLR2 is indicated as an important factor in HSV-2 induced cellular activation (52, 54) we examined the effects inhibition of the TRL2 pathway with an antagonistic TLR2 ligand had on the HIV infection. Our results exclude the involvement of TLR2 signaling in the enhanced HIV infection (Supplementary Figure 5). Taking into account that several intercellular sensors involved in HSV infection, e.g., IFI16, and cGAS, which merge their signaling pathways by the activation of STING (28, 48, 55), we examined the effect of STING activation on HIV infection. The levels of HIV infection in DCs after exogenous and endogenous stimulation with cGAMP, an activator of STING via cGAS, were investigated. The exogenously delivered cGAMP had a minor effect on the level of HIV infection, whereas the endogenously delivered cGAMP, i.e., delivered to the cytosol via DOTAP, significantly enhanced the HIV infection to levels similar to HIV/HSV exposed DCs (Figure 6B). This indicated that the intracellular DNA sensing cGAS/STING pathway is one of the pathways involved in the HSV-2 mediated enhancement of HIV infection in DCs. HIV infection combined with endogenous cGAMP, i.e., cGAMP delivered to the cytosol, induced higher expression levels of IFN-β in DCs, compared to HIV alone and HIV in combination with exogenously delivered cGAMP (Figure 6B). The higher levels of IFN-β expressed by HSV/HIV-exposed DCs compared to the levels induced by HIV in combination with endogenous cGAMP indicates that other signaling pathways/factors may be involved in the antiviral response in addition to the cGAMP/STING pathway. Noteworthy, the high IFN-β levels induced by the dual exposure did not hinder HIV infection in DCs as both cGAMP and HIV/HSV exposure induced high levels of HIV gag (Figure 6B).

### Dual HSV/HIV Exposure of DCs Activated and Subsequently Decreased the Protein Levels of Cytosolic DNA Sensors and Factors Involved in Their Signaling Pathways

**Figure 7:**
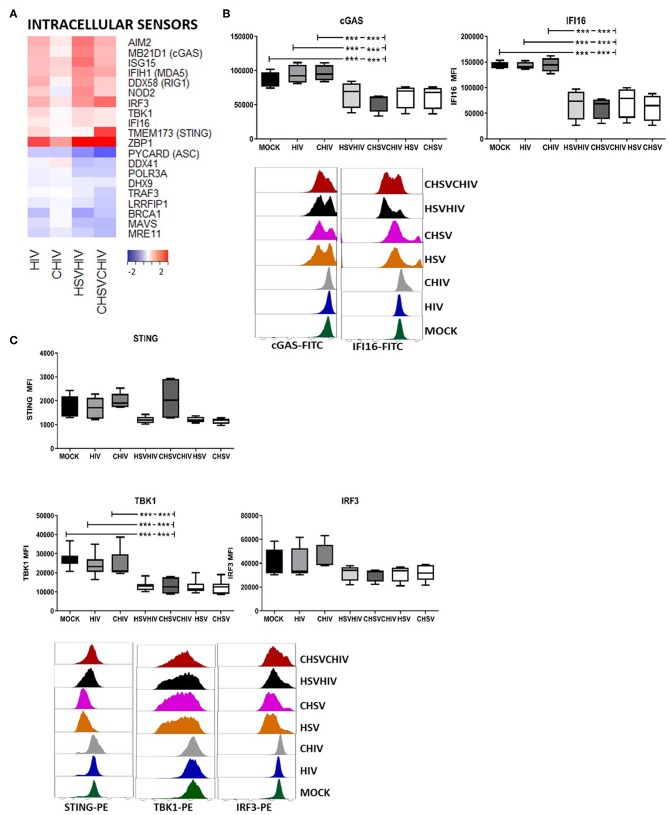
HSV-2 HIV infection of DCs induced the activation and degradation of the cytosolic DNA sensors. Dendritic cells were exposed to free HSV-2 (HSV) or complement opsonized virus (CHSV) for 2 h then infected with HIV or complement opsonized HIV (CHIV) for 22 h. **(A)** Heat map over antiviral factors significantly up or down regulated in one or several of the HIV, CHIV, HSV/HIV, or CHSV/CHIV DC groups compared to mock treated DCs by the RNA seq. **(B)** The levels of IFI16, and cGAS in the DCs from the different infection conditions by staining the cells and analyzed by flow cytometry. **(C)** The levels of STING, TBK1, and IRF3 in the DCs from the different infection conditions by staining the cells and analyzed by flow cytometry. ***p < 0.0005. *N* = 5–8.

The effects of HSV-2 on the intercellular sensors in DCs were assessed. The transcriptome analysis showed that the expression of cytosolic DNA and RNA sensors AIM2, MB21D1 (cGAS), IFIH1, DDX58 (RIG1), and ZBP1 (DAI) were all highly elevated, and IFI16 was slightly increased in DCs exposed to HIV alone and in dual exposed DCs. The expression of MAVS, DDX41, and DHX9 were decreased or unaffected by the different HIV and HSV/HIV conditions (Figure 7A). Several viruses including herpes viruses have the ability to regulate factors in the cellular innate and adaptive responses by the interference with signal transduction or degradation of proteins. For instance, HSV1 ICP27 inhibits the type I IFN expression in human macrophages by interaction with STING/TBK1 (56). Lytic reactivation of Kaposi's sarcoma-associated herpesvirus induces the degradation of IFI16 (25) and STING is degraded by a dengue virus protease (57). Moreover, stimulation of innate signaling pathways, e.g., STING, TBK1, and IRF3, can lead to protein downregulation post-pathogen activation of antiviral responses (58–60). In light of this, we assessed the protein levels of several DNA sensors and signaling molecules in DCs exposed to HIV, HSV, or HIV/HSV, respectively, and found that HSV alone and dual virus exposure significantly decreased the expression of the DNA sensors IFI16, and cGAS, both activators of the STING pathway, clearly indicating that this was foremost a HSV derived effect (Figure 7B). There were no significant differences in the effects seen with or without opsonization. So next, we examined the protein expression of factors in the STING pathway and found no significant effect on STING protein, even if there was a clear trend for decreased STING protein for HIV/HSV, but the STING downstream factor TBK1 was significantly decreased and a there was a trend of decreased expression for IRF3 in the dual exposed DCs compared to HIV alone (Figure 7C). Taken together, these findings indicate that proteolytic degradation of these proteins is induced foremost by HSV and may occur in DCs exposed to HSV-2, either as direct effects of viral proteins and/or due to viral activation of pathways involving these factors that subsequently leads to their degradation (58).

### Activation of the STING Pathway Was Needed for the Enhanced HIV Infection of DCs Induced by HSV-2

**Figure 8:**
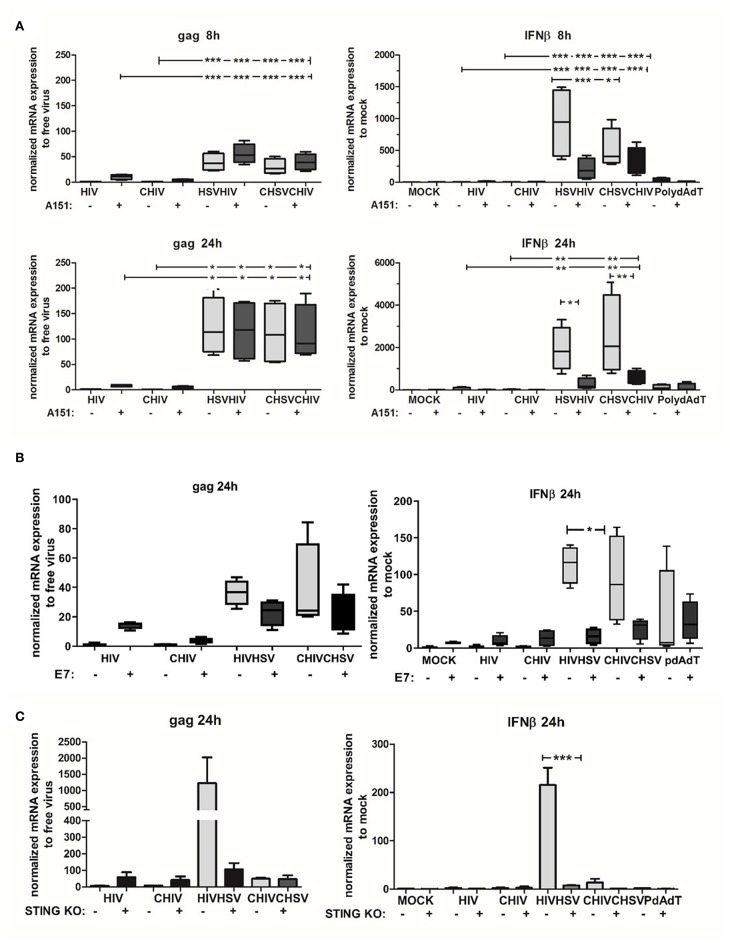
The STING pathway was involved in the enhanced HIV infection of HSV-2 exposed DCs. IFI16/cGAS inhibitory oligonucleotide A151 was delivered intracellularly by DOTAP to DCs. Cells were then exposed to free HSV-2 (HSV) or complement opsonized virus (CHSV) for 2 h before infection with HIV or complement opsonized HIV (CHIV) for **(A)** 6 h **(B)** or 22 h. **(B)** STING protein inhibitor HPV 18 E7 proteins was delivered intracellularly by DOTAP to DCs. Cells were then exposed to free HSV or CHSV for 2 h before infection with HIV or CHIV for 22 h. HIV gag transcript and IFN-β expression were evaluated by qPCR. **(C)** THP1 cells with STING knockout or wildtype were exposed to free HSV-2 or complement opsonized virus (CHSV) for 2 h then infected with HIV or complement opsonized HIV (CHIV) for 22 h. HIV gag transcript and IFN-β expression were evaluated by qPCR. HIV gag data were normalized to free virus set as 1 and IFN-β data were normalized to mock set as 1. *p < 0.05; **p < 0.005; ***p < 0.0005. *N* = 5–8.

The STING pathway is activated by upstream DNA sensors such as cGAS and IFI16, both implemented in the cytosolic recognition of HSV DNA (27, 61). In addition, recent data point to a collaboration between IFI16 and cGAS in the activation of STING (62), with distinct roles for that IFI16 and cGAS in antiviral responses against herpesviruses (63). Noteworthy, other studies state that cGAS is the major dsDNA sensor responsible for activation of the STING/TBK/IRF3 pathway (29, 64). Here we assessed the role of cGAS/IFI16 in the enhanced HIV infection in DCs conditioned by HSV-2 by using the inhibitory oligo deoxynucleotide A151 (65). Initially, intracellular A151 was assumed to inhibit IFI16 but recent findings established that it also affects cGAS activity (65, 66). The inhibitory oligo deoxynucleotide A151 slightly increased HIV infection assessed as gag transcript at the early time point (8 h) in DCs exposed to HIV and HSV/HIV. At 24 h there were no differences between A151 treated and untreated dual exposed DCs (Figure 8A). Interestingly, the inhibitory oligo deoxynucleotide A151 decreased the mRNA expression of IFN-β in the HSV/HIV conditions at both 8 and 24 h (Figure 8A). To confirm the cytosolic A151 result, we also tested the effect of cytosolic delivery of the herpes protein UL83, which has the ability to interfere with IFI16 (67) and cGAS functions (68), in DCs exposed to HIV or HSV/HIV. The UL83 showed the same profile as A151 for HIV gag and IFN-β mRNA transcript expression (Figure 8; Supplementary Figure 6). The data indicated that inhibition of cGAS/IFI16 with inhibitory oligo deoxynucleotide A151 or with UL83 was not enough for suppression of the enhanced HIV infection but succeeded to lower, but not fully suppress, the IFN-β response so we could not rule out residual activity of these pathways in the DCs. To further explore the role of the STING pathway in the enhanced infection and elevated antiviral response in the dual exposed DCs, we targeted STING protein using a recombinant HPV 18 E7 protein known to inhibit this pathway by binding and blocking STING (69, 70). The intracellularly delivered E7 succeeded to decrease the gag transcript expression and the IFN-β in the dual infected DCs. The decrease was however not significant for gag but a trend could be seen (Figure 8B). Next, to verify and strengthen the role of STING, we assessed the role of STING pathway using a THP1 cell line lacking sting due to STING knockout with siRNA and found a decreased gag expression in HIV/HSV exposed cells but no clear evidence for this in CHSV/CHIV (Figure 8C), which could be due to the THP1's low level of CR3. Taken together, our findings indicate that the STING pathway is involved in the enhanced HIV infection in HSV-2 exposed DCs.

### The Enhanced HIV Infection in DCs Conditioned With HSV-2 Was Probably Due to the Decreased Levels of HIV Regulatory Factors as a Consequence of the Activation of the cGAS-STING Pathway

**Figure 9:**
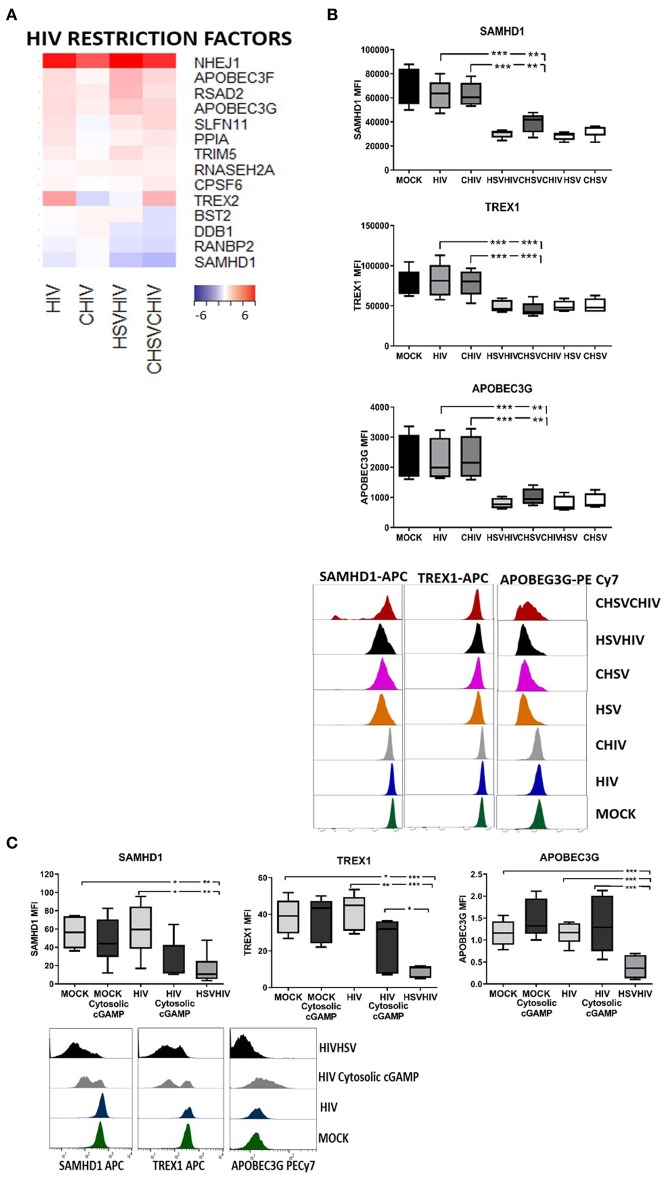
STING pathway activation in the dual infected DCs decreased protein levels of several HIV restriction factors. Dendritic cells were exposed to free HSV-2 or complement opsonized virus (CHSV) for 2 h then infected with HIV or complement opsonized HIV (CHIV) for 22 h. **(A)** Heat map from the RNA seq transcriptome data of intracellular sensors significantly up or down regulated in one or several of the HIV, CHIV, HSV/HIV, or CHSV/CHIV infection conditions compared to mock treated DCs. **(B)** The levels of SAMHD1, ABOBEC3G, and TREX1 in the DCs from the different infection conditions were assessed by staining the cells and analyzed by flow cytometry. **(C)** cGAMP was given exogenously or delivered intracellularly via DOTAP to the DCs, following this were the DCs left unexposed or exposed to together with HIV for 16 h. In addition, one group of DCs were exposed to HSV for 2 h followed by HIV or CHIV for 16 h. The levels of SAMHD1, ABOBEC3G, and TREX1 were assessed by staining the cells and analyzed by flow cytometry. *p < 0.05; **p < 0.005; ***p < 0.0005. *N* = 5–8.

The transcriptome profiles clearly demonstrated combinatory effects of the HSV-2 and HIV exposure on many of the master regulators of HIV infection in DCs including SAMHD1, TREX, APOBEC3G, PPIA (cyclophilin A), and BST2 (tetherin) (Figure 9A). The gene expression of the majority of HIV regulatory factors was increased in DCs infected with HIV alone or in combination with HSV-2 compared to control, whereas SAMHD1 expression was decreased. In addition, BST2 was slightly decreased in the CHIV/CHSV group. HSV-2 infection can modulate the expression of several proteins (25, 71) and the activation of antiviral and innate pathways might influence the protein expression and levels so we examined the effect the HSV-2 exposure had on the protein levels of the HIV regulators in DCs. The protein levels of SAMHD1, APOBEC3G, and TREX1 were significantly lower in the single HSV and CHSV and in dual HSV/HIV and CHIV/CHSV exposed DCs compared to mock treated cells. HIV and CHIV had no or minimal effect on these factors (Figure 9B). Next, we assessed if the decrease in HIV regulatory factors was dependent on intracellular activation of the cGAS-STING pathway by HSV-2 derived dsDNA, by using cGAMP, which is produced after dsDNA activation of cGAS (61, 72). The DCs exposed to both cytosolic cGAMP and HIV had lower protein levels of both SAMHD1 and TREX1 compared to DCs exposed to HIV alone (Figure 9C). In the case of APOBEC3G, the expression was not affected by cGAMP indicative of a more complex activation and/or proteolysis induced by HSV-2 derived factors compared to cGAMP alone (Figure 9C). These findings demonstrate that the cGAS-STING pathway, activated by intact HSV-2 DNA, contributes to HSV-2 mediated enhancement of HIV infection in DCs, by decreasing the amount of several key HIV regulatory factors.

## Discussion

A deeper understanding of factors influencing HIV infection is of significant importance, and pre-existing infection and/or inflammation in the genital mucosa has been shown to increase the risk for HIV acquisition. In the case for HSV-2 there is a three-fold higher risk to acquire HIV infection (2, 9, 14). During the primary HIV infection of mucosa, HSV-2, both infectious and neutralized viruses, and debris from dying infected cells, will influence the local innate and adaptive immune responses. DCs located in the mucosa are among the first cells targeted by HIV. Here, we aimed to establish the effects exerted by HSV-2 conditioning of DCs prior to HIV infection. We found a higher susceptibility and level of productive HIV infection in DCs exposed to HSV-2, which was dependent on structurally intact HSV-2 DNA in DCs. Noteworthy, the high levels of IFN-β production induced in the DCs by the HSV-2 exposure failed to control the infection as IFN-β increased in parallel with increased HIV infection in the dual exposed DCs. The HSV-2's effect on HIV replication in DCs was not dependent on TLR2, TLR3, or IFI16, all three PRRs known to be activated by herpes virus (55), but rather on the activation of the cGAS-STING pathway and subsequent decreased protein levels of several key regulators of HIV infection, i.e., TREX1, SAMHD1, and ABOBEC3G, which could be due to proteolytic degradation.

DCs located in the mucosa are regarded as a target for HIV-1 as has been shown in macaques infected by SIV and in cervix and foreskin mucosa *in vitro* models (73–75). The initial host-virus interaction in the mucosa also includes the activation of complement system (19) and both the innate and adaptive immunity activated is influenced by the complement system and complement can be involved in the pathogenies of chronic diseases (76). The virions transferred during sexual transmission to DCs should be complement and/or complement antibody opsonized particles seeing the exposure to seminal fluid and or cervix secretions (19). The result in our study using monocyte derived DCs, which we believe to be a good model for *in vivo* tissue/mucosal DCs, seeing similar results for free and complement opsonized HIV-1 infection and for HSV-2 infection in cervical mucosal DCs [(75) and Svanberg et al. work in progress]. The monocyte derived DCs we used appear to be similar to the newly defined CD11c+ epidermal DCs, which is a target for HIV infection (77). Epidermal CD11c+ DCs and Langerhans cells might play an important role in the sexual transmission of HIV-1 (8, 77). The fact that merely a few percentages of DCs exposed to HIV and HSV-2 are productively infected with both viruses (8), clearly indicates that the enhanced HIV infection of DCs only required exposure to HSV-2 virions or virus-derived DNA. This will establish a context where a preexisting HSV-2 infection attracts and provides more target cells for the HIV, e.g., CD4 T cells and DCs (2, 8, 9), as well as an environment with micro areas with HSV-2, proinflammatory cytokines, and HSV-2 DNA released by dying cells. Taken together all these factors will precondition the mucosal DCs and render them more susceptibility to productive HIV infection.

The initial transcriptome analysis demonstrated that all infection conditions had distinct expression profiles with some similarities between the conditions with free viruses, i.e., HIV and HIV/HSV, and between complement opsonized viruses, i.e., CHIV and CHIV/CHSV. Numerous pathways involved in antiviral and inflammatory responses were highly affected in the DCs infected with free HIV or complement opsonized or non-opsonized HSV/HIV, whereas complement opsonized viruses in general activated lower amount of pathways. The efficient suppression of DC activation by complement opsonized HIV should foremost be due to the suppression of innate and adaptive pathways mediated by the complement receptor 3 (CR3) signaling engaged by the inactivated C3b fragment on the viral surface (20). The preconditioning of DCs with HSV-2 followed by HIV infection clearly potentiated the activation of DCs compared to the HIV infection alone. This should be dependent primarily on the strong and long-lasting activation elicited by HSV-2 seeing that HIV alone give rise to a weak and more transient activation of the DCs.

There was a massive transcriptional activation of inflammatory and antiviral factors in the HIV/HSV exposed DCs however this was not fully reflected on the protein level with no changes or even decreased levels for some proteins. This is in accordance to our previous observation for DCs infected with HSV-2 (18). There could be multiple reasons behind this discrepancy such as post-transcriptional modification, degradation by HSV-2 derived proteins (25, 56) and/or a targeted proteolytic degradation of proteins involved in the cellular processes owing to viral activation of cellular stress such as autophagy and post-pathogen activation downregulation of factors. Herpes viruses have a wide range of strategies to evade innate immune sensing and to enhance their own survival. Common strategies utilized by herpes viruses are to inhibit or degrade proteins involved in the antiviral immune responses (25, 48, 78). Several HSV proteins and viral-non-coding RNAs can modulate protein expression, for instance the HSV encoded UL41 protein is proposed to have the ability to trigger a fast and selective shut-off of the host cell's protein synthesis for several proteins including viperin, and tetherin (79–82).

Multiple PRRs have been shown to be involved in the cellular recognition and defense against herpesviruses (55), when we tested TLR2 and TLR3 we found none of these TLRs to be involved in the elevated HIV infection seen in the HSV-2 conditioned DCs. Recent reports have instead indicated the activation of the STING pathway and its important role in the antiviral defense against HSV (83, 84). The STING singaling cascade can intersect both the TBK1/IRF3 and the NFκB pathway (85–87). Several DNA sensors has been implicated in the activation of STING, such as IFI16, DDX41, and cGAS, but accumulative evidence has made it clear that cGAS-sensing is required in most cell types. This is true even if there is a cooperation in the activation of STING between IFI16 and cGAS in keratinocytes (62) and IFI16 promote the cGAS production of cGAMP in human macrophages (72). All our data point toward activation of the cGAS STING pathway in the dual infected DCs. When it comes to the elevated HIV infection, we can rule out the involvement of IFI16 since blocking this factor failed to decrease the HIV infection whereas direct targeting of STING succeeded to do the same. Then again, we cannot rule out an involvement of IFI16 in sensing the dual infection and the cooperation of this sensor with cGAS in the activation of the antiviral IFN-β responses since block of IFI16 decreased the IFN-β response. The important role of IFI16 in sensing intracellular DNA has previously been shown in human macrophages, by means of enhancing production and function of cGAMP (72).

The HIV infection of DCs is tightly regulated and limited by an array of antiviral factors such as SAMHD1, APOBEC3G, TRIM5α, SLFN11, and IFITMs that exert their effects at different steps in the HIV replication cycle (88–90). These factors control or suppress HIVs ability to induce a productive infection with SAMHD1 as the major HIV suppressive factor (90, 91). SAMHD1 inhibits the early steps of HIV infection by limiting the viral cDNA synthesis by depleting deoxynucleotide triphosphates (dNTPs) (90) and SAMHD1 degradation by HIV-2 VPX give rise to enhanced infection of DCs and production of type I IFNs (91). HIV uses the host 3′-repare exonuclease 1 (TREX1), the most abundant 3′-5′ DNase in the cells, to bind and degrade excess cytosolic HIV-1 dsDNA and thereby limit the type I IFN response (88). In addition to restricting HIV infection, SAMHD1 and TRIM5α have also been shown to be involved in regulating the HSV-1 infection by limiting the DNA replication (92, 93). We found a high degree of proteolytic degradation of several of the HIV-1 restriction factors including SAMHD1, IFI16, TREX1, and APOBEC3G (88–90) in the dual exposed DCs. The suboptimal levels of several key restriction factors can explain the loss of the DCs' control of the HIV infection and the enhanced productive HIV infection in the DCs. Of interest, the activation of the cGAS-STING pathway in DCs in parallel with HIV exposure induced the degradation of SAMHD1 and TREX1, but not of APOBEC3G. Moreover, the intracellular activation of cGAS-STING pathway with cGAMP gave similar effects on HIV replication and cytokine production as the preconditioning with HSV-2, indicating an essential role for this pathway in the HSV-2 cellular modulation of DC functions. The activation of the STING pathway in the DCs exposed to both HIV and HSV-2 should subsequently lead to accumulation of more nucleotides in the cytosol due to lower levels of the 3′-5′ exonuclease TREX1 and the phosphohydrolase SAMHD1, which should support the viral replication. The lack of APOBEC3G degradation by the exogenous cGAMP activation of STING indicate that other HSV-2 induced processes/factors were involved in this and exact which factors remains to be established but could involve HSV derived factors or factors activated by the virus exposure. For instance, the finding by Marsden et al. that the TNF produced by HSV-2 exposed DCs was involved in the enhanced HIV infection of bystander DCs (8), indicates that TNF might be one of the factors involved in promoting the HIV infection in DCs.

Numerous of the cytosolic DNA sensors and cofactors, i.e., IFI16, cGAS, STING, TBK1, and IRF3, important for the recognition of viral PAMPs were also targeted for degradation in the dual HIV and HSV-2 exposed DCs but not in HIV exposed DCs, demonstrating direct and/or indirect involvement of the HSV-2 in this process. The decrease of several proteins involved in the STING pathway, e.g., IFI16, cGAS, STING, TBK1, and IRF3 was probably due to viral activation of the STING pathway (58, 94) and/or direct degradation by viral derived proteins (95). To dissect this further, future studies exploring phosphorylation and localization of these proteins are needed. Several DNA virus proteins can cleave or degrade DNA sensors. For instance, the dsDNA sensor IFI16, which activates IFN-β responses and forms an inflammasome, is degraded in HSV-1 infected cells by ICP0 proteasomal targeting (48) and in cells with lytic Kaposi's Sarcoma-Associated Herpesvirus infection (25). STING and cGAS are also common targets for DNA virus proteins that degrade these factors or interfere with their downstream signaling (57, 70, 96–98). In the dual exposed DCs the levels of cGAS were lower compared to the non-infected or single HIV infected cells and this should lower the production of cGAMP and the activation of STING as a result of the cellular regulation and control of this factor after activation (56). Several viruses are sensed and regulated by the STING pathway including HSV-1, HSV-2, and HIV-1 (29, 56). HIV-1 DNA can be sensed by cGAS and the STING pathway (29), however, this is counteracted and prevented by the HIV-1's capsid by recruitment of cellular proteins, e.g., cyclophilin A and TRIM5α (99). HSV-1 counteracts the STING-cGAS pathway by several proteins including ICP27, which can inhibit the type I IFN expression by targeting the STING signaling complex (56). It is clear that both HIV and HSV-2 are regulated by the STING pathway and have evolved strategies to establish infection by dampening the STING activation and subsequent type I IFN responses.

We found and have previously shown (20) that in the monocyte derived DCs, exposure to HIV-1 alone do succeed to induce a burst of low level of IFN-β, this response is transient and weak compared with many other viruses, including HSV-2 (18). We think this finding is in accordance to previous studies by Harman et al. and Gringhuis et al. that demonstrate that HIV-1 has the ability, by several mechanisms to interfere with IFN-β production and avoid antiviral host defenses (100–102). The dual exposure induced high level of the antiviral factor IFN-β and subsequent ISG such as MX1 and MX2 in the DCs via the HSV-2 DNA activation of the STING pathway. The high level of IFNβ production in dual free and complement oponized HIV/HSV exposed DCs failed to block the HIV infection but it is clear that it subdued the infection, seeing that the HIV infection was further elevated in DCs where the IFN-β was suppressed by inhibition of the DNA sensor IFI16. This demonstrated a balancing act by the STING pathway playing a part both in the enhanced HIV infection but also in the control of the level of the infection by its activation of the type I IFNs. We have previously established that HIV-1 can induce IFN-β production in DCs in an IRF1/7 dependent way, whereas the complement opsonized HIV activated IRF3, but this gave no or very low IFN-β production (20). This is probably due to the CR3 induced suppression by the complement opsonized virus and that CR3 suppression of IFN-β is overridden in the strong activation the HSV-2 induce in the DCs.

We clearly demonstrate that the HSV-2 dependent augmentation of HIV infection required intact HSV-2 dsDNA, but not an active HSV DNA replication. The cellular support of productive HIV infection should be due to the regulation and proteolytic degradation of HIV restriction factors, i.e., SAMHD1, APOBEC3G, IFI16, and TREX1. The mechanism responsible for the proteolytic degradation of SAMHD1, TREX1 and APOBEC3G proteins seems to be facilitated by the activation of the STING pathway, which has to our knowledge not been described to date, and the effect of one or several of the HSV-2 proteins. The degradation of several of the HIV regulatory factors seen when we activated STING via cGAMP-cGAS pathway by addition of exogenous cGAMP, support the role of the STING pathway in the degradation of these proteins in the dual HIV HSV exposed DCs. Furthermore, the antiviral and inflammatory pathways activated by HIV alone or dual HIV/HSV exposure were differently shaped in the presence or absence of complement molecules. These findings demonstrated that the HSV-2 intracellular reprogramming enhances the ability of HIV to infect DCs and provides a conditioned microenvironment favorable for HIV transmission and the establishment of infection.

## Data Availability Statement

The datasets generated for this study can be found in the GEO—accession GSE140612. Other raw data supporting the conclusions of this manuscript will be made available by the authors, without undue reservation, to any qualified researcher.

## Ethics Statement

The studies involving human participants were reviewed and approved by Swedish ethical review board Ethical Permits M173-07, and M75-08/2008. Written informed consent for participation was not required for this study in accordance with the national legislation and the institutional requirements.

## Author Contributions

EC, CS, and ML designed research. EC, RE, CS, PB, MK, JH, and SN performed research. KE contributed new reagents and analytic tools. KO, EC, RE, CS, KE, ES, and ML analyzed data. EC, KE, CS, ES, and ML wrote the paper. ML supervised the project.

### Conflict of Interest

The authors declare that the research was conducted in the absence of any commercial or financial relationships that could be construed as a potential conflict of interest.
