# Supplementary material for: HSV-2 Cellular Programming Enables Productive HIV Infection in Dendritic Cells
Source: Front Immunol. 2019 Dec 6;10:2889. doi: 10.3389/fimmu.2019.02889 (PMC6909011; doi:10.3389/fimmu.2019.02889)
Supplement: Supplementary file 1 [file Data_Sheet_1.pdf]

Figures Supplementary figures and legend

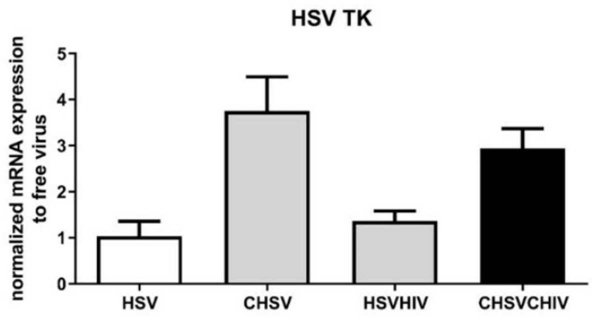

Supplementary Figure 1. TK expression was increased in DCs exposed to complement opsonized HSV-2.

DCs were exposed to free HSV-2 (HSV) or complement opsonized HSV-2 (CHSV) for 2h and thereafter exposed to HIV for 24h. mRNA levels of the viral induced expression of TK was measured using qPCR. HSV-2 TK transcript data were normalized to HIV set as 1. N=5.

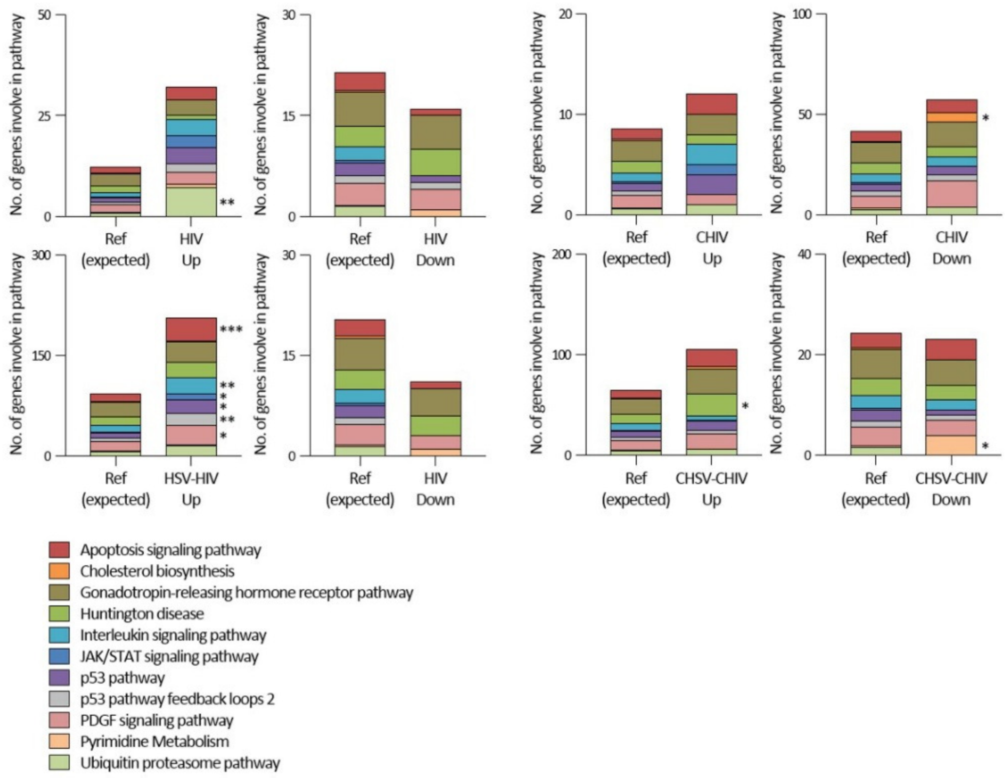

Supplementary Figure 2. Cellular programing of dendritic cells by HIV single and HIV/HSV dual infection.

Dendritic cells were exposed to HSV-2 (HSV) or complement opsonized virus (CHSV) for 2h then infected with HIV or complement opsonized HIV (CHIV) for 24h. Whole transcriptome sequencing was performed. Gene enrichment analysis of genes significantly upregulated one fold or higher with $p < 0.01$ or down regulated one fold or higher with $p < 0.01$. GO enrichment analysis was done with PANTHER pathways data set. Terms with statistical significance in any of gene list are shown as stacked bar graph. *: $p < 0.05$, **: $p < 0.005$, ***: $p < 0.0005$. Y-axis = number of listed genes involving in indicated PANTHER pathway. Ref = expected gene number from reference (whole human genes in database). N=5.

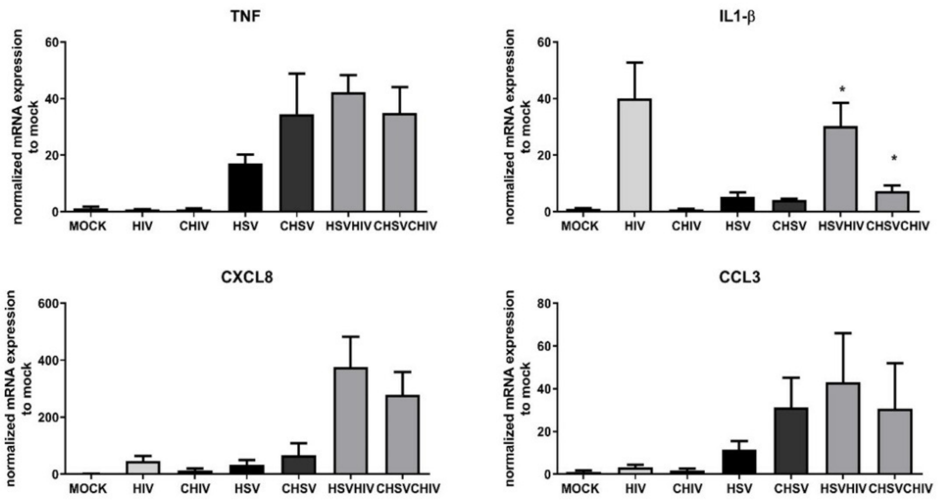

Supplementary Figure 3. HSV-2 and HIV are capable of inducing inflammatory factors in DCs.

DCs were exposed to free HSV-2 (HSV) or complement opsonized HSV-2 (CHSV) for 2h before infection with HIV or complement opsonized HIV (CHIV). mRNA levels of the inflammatory cytokines and chemokines TNFα, IL-1β, CXCL8 and CCL3 was measured using qPCR. Transcript data were normalized to HIV expression set as 1. *: $p < 0.05$, **: $p < 0.005$, ***: $p < 0.0005$. N=3-5.

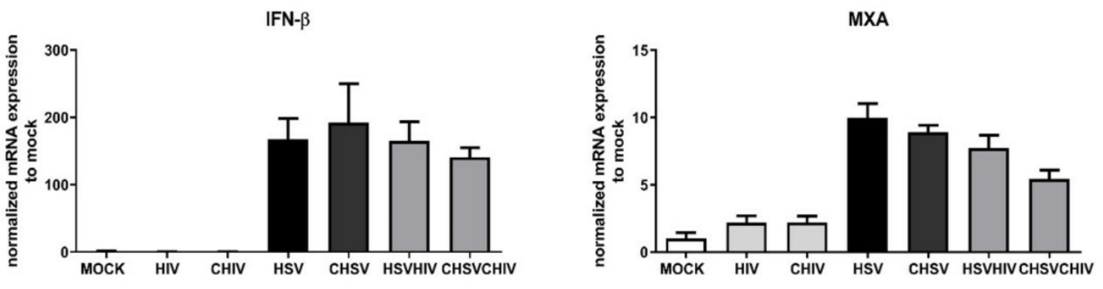

Supplementary Figure 4. HSV-2 is the main inducer of antiviral factors in DCs.

DCs were exposed to free HSV-2 (HSV) or complement opsonized HSV-2 (CHSV) for 2h before infection with HIV or complement opsonized HIV (CHIV). mRNA levels of the anti-viral factors IFN-β and MXA was measured using qPCR. Transcript data were normalized to HIV expression set as 1. *: $p < 0.05$, **: $p < 0.005$, ***: $p < 0.0005$. N=3-5.

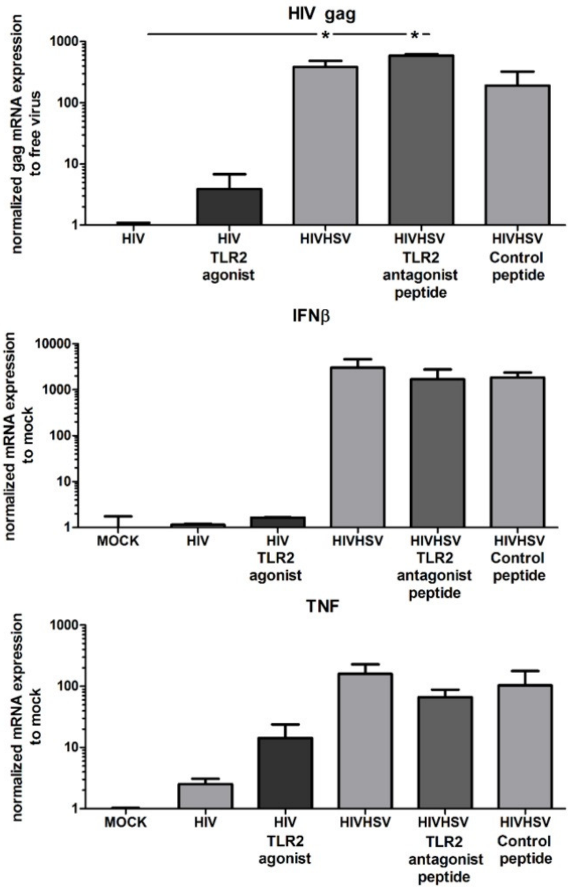

Supplementary Figure 5. TLR2 activation was not required for the enhanced HIV infection in HSV-2 exposed DCs.

Immature DCs were exposed to TLR2 agonist, TLR2 antagonistic peptide or control peptide alone or in combination with HIV for 16h or exposed to HSV-2 (HSV) for 2h followed by HIV for 16h. HIV gag transcript, TNF, and IFN-β expression were evaluated by PCR. HIV gag transcript data were normalized to HIV set as 1 and TNF, IFN-β data were normalized to mock set as 1. * p<0.05 ** p<0.005 *** p<0.0005. N=5-8.

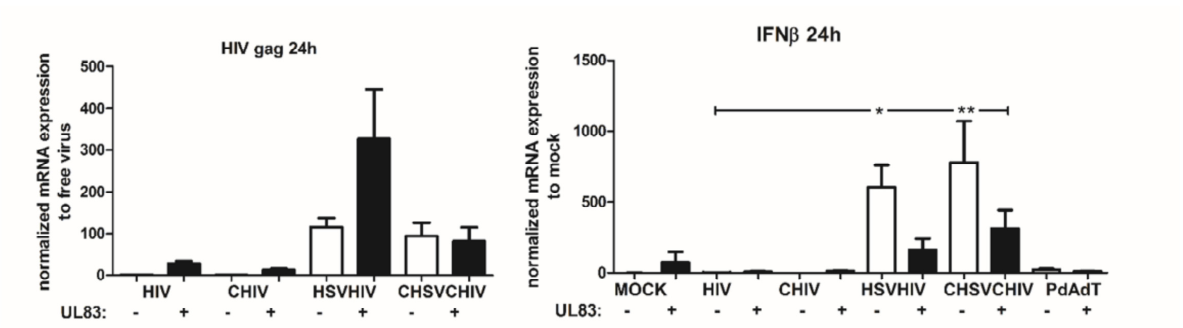

Supplementary Figure 6. The block of cGAS/IFI16 was not sufficient for the lowering the elevated HIV infection of HSV-2 exposed DCs.

UL83 protein, an IFI16/cGAS inhibitor, was delivered intracellularly by DOTAP to DCs. Cells were then exposed to free HSV-2 (HSV) or complement opsonized virus (CHSV) for 2h before infection with HIV or complement opsonized HIV (CHIV) for 22h. HIV gag data were normalized to free virus set as 1 and IFN-β data were normalized to mock set as 1. * p<0.05 ** p<0.005 *** p<0.0005. N=5-8.
